# Global prevalence and species diversity of tick-borne pathogens in buffaloes worldwide: a systematic review and meta-analysis

**DOI:** 10.1186/s13071-023-05727-y

**Published:** 2023-03-30

**Authors:** El-Sayed El-Alfy, Ibrahim Abbas, Rana Elseadawy, Somaya Saleh, Bassem Elmishmishy, Shimaa Abd El-Salam El-Sayed, Mohamed Abdo Rizk

**Affiliations:** 1 Parasitology Department, Faculty of Veterinary Medicine, Mansoura University, Mansoura, 35516 Egypt; 2 Department of Biochemistry and Chemistry of Nutrition, Faculty of Veterinary Medicine, Mansoura University, Mansoura, 35516 Egypt; 3 Department of Internal Medicine, Infectious and Fish Diseases, Faculty of Veterinary Medicine, Mansoura University, Mansoura, 35516 Egypt

**Keywords:** Tick-borne pathogens, Buffaloes, Meta-analysis, *Babesia*, *Theileria*, *Anaplasma*

## Abstract

**Background:**

Buffaloes are important contributors to the livestock economy in many countries, particularly in Asia, and tick-borne pathogens (TBPs) commonly infect buffaloes, giving rise to serious pathologies other than their zoonotic potential.

**Methods:**

The present investigation focuses on the prevalence of TBPs infecting buffaloes worldwide. All published global data on TBPs in buffaloes were collected from different databases (e.g., PubMed, Scopus, ScienceDirect, and Google Scholar) and subjected to various meta-analyses using OpenMeta[Analyst] software, and all analyses were conducted based on a 95% confidence interval.

**Results:**

Over 100 articles discussing the prevalence and species diversity of TBPs in buffaloes were retrieved. Most of these reports focused on water buffaloes (*Bubalus bubalis*), whereas a few reports on TBPs in African buffaloes (*Syncerus caffer*) had been published. The pooled global prevalence of the apicomplexan parasites *Babesia* and *Theileria*, as well as the bacterial pathogens *Anaplasma*, *Coxiella burnetii*, *Borrelia*, *Bartonella*, and *Ehrlichia* in addition to Crimean-Congo hemorrhagic fever virus, were all evaluated based on the detection methods and 95% confidence intervals. Interestingly, no *Rickettsia* spp. were detected in buffaloes with scarce data. TBPs of buffaloes displayed a fairly high species diversity, which underlines the high infection risk to other animals, especially cattle. *Babesia bovis*, *B. bigemina*, *B. orientalis*, *B. occultans* and *B. naoakii*, *Theileria annulata*, *T. orientalis* complex (*orientalis/sergenti/buffeli*), *T. parva*, *T. mutans*, *T. sinensis*, *T. velifera*, *T. lestoquardi*-like, *T. taurotragi*, *T.* sp. (buffalo) and *T. ovis*, and *Anaplasma marginale*, *A. centrale*, *A. platys*, *A. platys*-like and “*Candidatus* Anaplasma boleense” were all were identified from naturally infected buffaloes.

**Conclusions:**

Several important aspects were highlighted for the status of TBPs, which have serious economic implications for the buffalo as well as cattle industries, particularly in Asian and African countries, which should aid in the development and implementation of prevention and control methods for veterinary care practitioners, and animal owners.

**Graphical Abstract:**

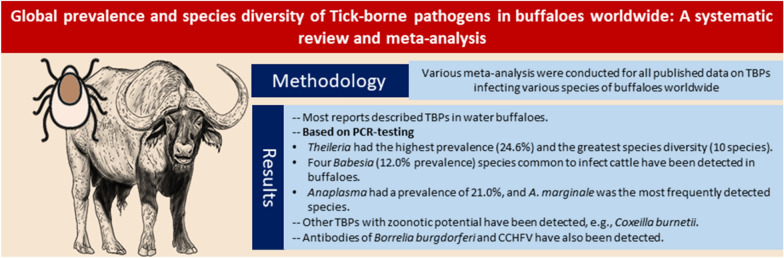

**Supplementary Information:**

The online version contains supplementary material available at 10.1186/s13071-023-05727-y.

## Background

Along with cattle, buffaloes are important members of the subfamily Bovinae, and include various species, of which water buffaloes (*Bubalus bubalis*) are the most important and widely distributed worldwide over 77 countries on five continents, with a population exceeding 200 million [1, 2]. Water buffaloes (also known as domestic water buffalo or Asian buffalo) have two genetically distinct subspecies: river buffalo (81.5% of the global population) and swamp buffalo (18.5%) [2, 3]. The former is native to the Indian subcontinent [4] and has spread west, across southwestern Asia, Egypt, and Anatolia, and reached the Balkans and the Italian peninsula [5, 6]. Swamp buffaloes are predominant in Southeast Asia and Australia [4, 5, 7, 8]. The global water buffalo population is concentrated mostly in Asia (196 million) [4]. Smaller numbers are reared in Africa (3.4 million) and South America (2.0 million). Another buffalo species, *Syncerus caffer* (the African buffalo), lives in sub-Saharan Africa [9]; however, its numbers have been severely reduced since the eighteenth century as a result of the combined effects of anthropogenic pressures (e.g., land conversion), disease outbreaks, and climatic changes [10]. Although water buffaloes are mostly reared for milk production, they also contribute to the global meat sector, with around 4.3 million tons annually [11]. In some regions, buffaloes serve as working animals in various agricultural fields.

Tick-borne diseases (TBDs) are significant factors limiting the development of livestock industries worldwide, resulting in annual economic losses that can be estimated in billions of dollars [12, 13]. For example, the annual economic loss due to TBDs infecting cattle in Tanzania has been estimated at US$ 364 million [14]. Tick-borne pathogens (TBPs) pose a significant threat to buffalo health and production other than their zoonotic risks. In recent years, much progress has been made in the characterization and taxonomic justification of TBPs infecting buffaloes worldwide. Nonetheless, studies detailing various epidemiological aspects of TBPs infecting buffaloes are scarce. The present study provides a systematic review and meta-analysis of the global published data on TBPs infecting buffaloes worldwide, which should be useful for interpreting the epidemiology of this important group of pathogens.

## Methods

### Search strategy

The international databases PubMed, Scopus, ScienceDirect, and Google Scholar were systematically searched for studies on TBPs infecting buffaloes, with no date limit. The search was refined by article language (English) and type (research articles). Various keywords were used for the search: ticks; tick-borne pathogens; tick-borne diseases; *Anaplasma*, anaplasmosis; *Babesia,* babesiosis; *Theileria*, theileriosis; CCHF, Crimean-Congo hemorrhagic fever; *Coxiella burnetii*, Q fever; *Ehrlichia*, ehrlichiosis; *Rickettsia*, rickettsioses; *Borrelia*, borreliosis. These keywords were used in combination with the word “buffaloes,” and connected using the Boolean operators “AND” and “OR.”

### Eligibility criteria

**Fig. 1:**
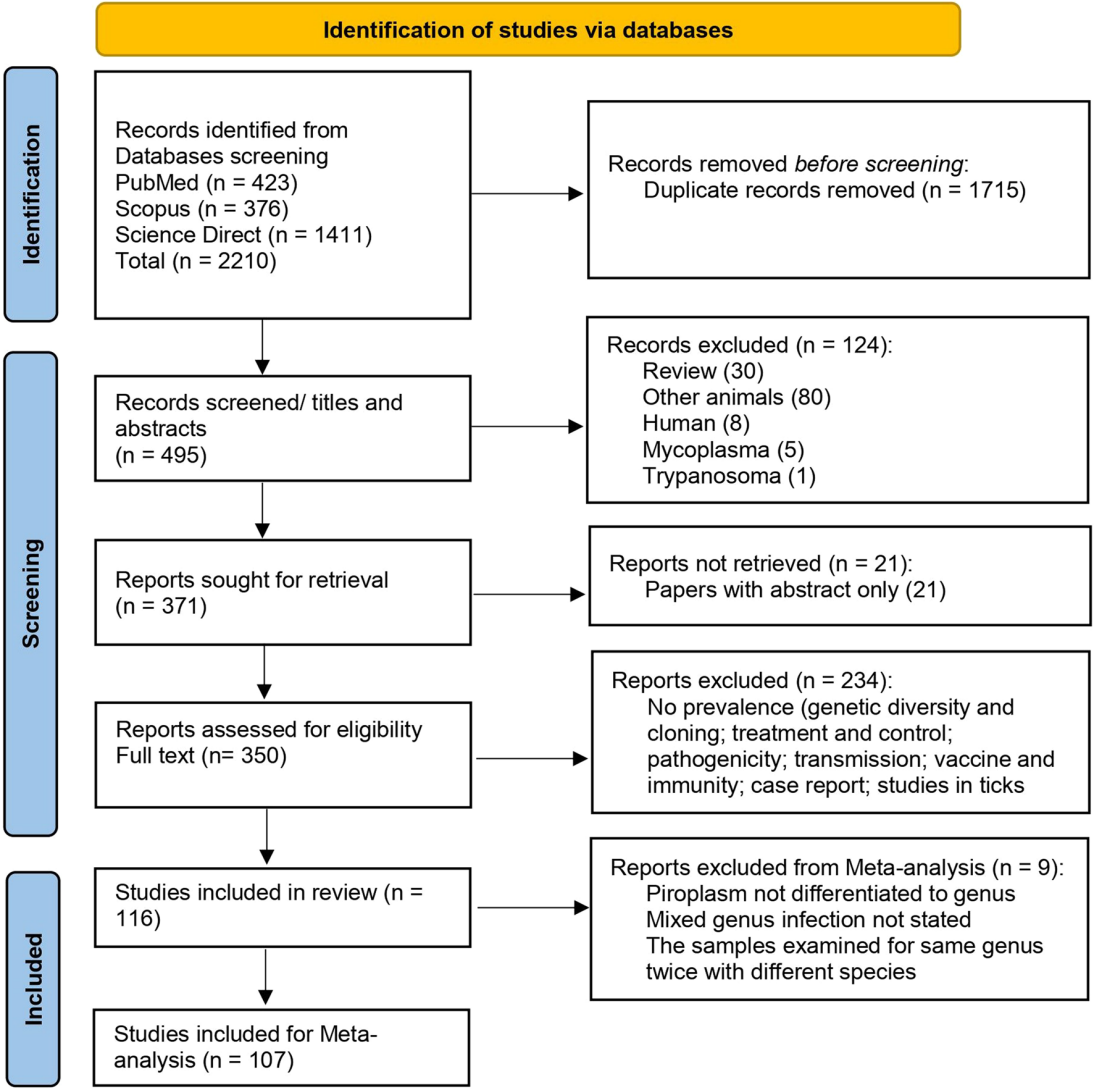
Flow diagram established according to PRISMA [Preferred Reporting Items for Systematic Reviews and Meta-Analyses] guidelines and displaying the search and selection methodology

The collected publications were screened for inclusion independently by two of the authors, and any article with disagreement was discussed with a third author (Fig. 1). Studies were considered eligible to be included in this meta-analysis when (1) the study investigated TBPs in samples from buffaloes, either water (*B. bubalis*) or African (*S. caffer*) species, and (2) the study defined the number of examined buffaloes and number of positives. Studies on TBPs in animals other than buffaloes or those with inadequate methodologies were considered ineligible, and articles of non-original contributions including reviews, book chapters, and seminars were also excluded.

### Data extraction

Data from eligible studies on TBPs of buffaloes were independently extracted and organized in a Microsoft Excel^®^ spreadsheet by two of the authors. Any disagreement was resolved by consensus. The following information was extracted: authors, publication year, study subregion/country, number of tested samples, number of positive samples, detection methods employed, and various TBPs detected as well as the genetic markers (where recorded). Authors of the included articles were not contacted for further information, and no data conversions were conducted in the present study.

### Meta-analysis

Data tabulated in the Excel spreadsheets were used for various meta-analyses conducted in the present study using OpenMeta[Analyst] software [15], and all analyses were conducted based on a 95% confidence interval (CI). The prevalence for various TBPs was calculated as “a pooled estimate” using the random-effects model associated with the DerSimonian–Laird method [16]. The heterogeneity between studies was estimated based on the *I*^*2*^ statistic. The heterogeneity values were considered high when *I*^2^ exceeded 50% [17, 18]. Subgroup analyses were conducted to detect the source of heterogeneity and included the variation in prevalence among various worldwide regions and the detection method. Publication bias was not assessed in the present study because it is not considered relevant for prevalence studies [19].

## Results and discussion

### Eligible studies

Data from 116 studies were found eligible to describe the TBPs infecting buffaloes worldwide, employing various detection methods [20–135]. Of these studies, 107 were used for meta-analysis (Fig. 1), and nine were not included [42, 69, 91, 103, 104, 108, 116, 119, 123] for the following reasons: the piroplasm was not identified even to the genus (*n* = 2), undefined mixed infections (*n *= 4), and double testing of the samples to identify two separate species of the same genus (*n* = 3). The country-wise distribution of these studies correlated with the number of reared buffaloes. The majority of these studies (*n* = 75) came from Asia [43–117], including India (21), Pakistan (13), China (10), Thailand (8), the Philippines (6), Vietnam (6), Iran (3), Sri Lanka (3), Iraq (1), Malaysia (1), Myanmar (1), Turkey (1), and Laos (1). Twenty-three studies were conducted in Africa [20–42], around two thirds (*n* = 15) of them came from Egypt [21–35], and a few studies were conducted in South Africa (3), Botswana (1), Kenya (1), Namibia (1), Tanzania (1), and Uganda (1). A few surveys (*n* = 12) were conducted on buffaloes from South America [124–135] including Brazil (9), Colombia (2), and Argentina (1). The lowest number (*n* = 4) of the eligible studies came from Europe [118–121]: Hungary (2), the Czech Republic (1), and Italy (1). Likewise, isolated surveys were conducted on buffaloes from North America (2), Cuba (1), and Mexico (1) (Additional file 1: Table S1). These studies comprised 145 datasets according to the detection method used, which included 57 datasets on *Babesia* spp., 48 on *Theileria* spp., 41 for *Anaplasma* spp., 15 for *Coxiella burnetii*, and 22 for miscellaneous TBPs (e.g., bacterial pathogens; *Borrelia*, *Bartonella*, *Ehrlichia*, and *Rickettsia* spp., and Crimean-Congo hemorrhagic fever virus [CCHFV]). Although data were divided into subgroups of investigated regions by continent, many studies did not include the buffalo species. As a result, data were not classified according to buffalo species, particularly African (*S. caffer*) and Asian (*B. bubalis*) buffaloes.

### Piroplasmids

Ticks can transmit a variety of *Babesia* and *Theileria* piroplasmids, which can cause serious infections as well as economic consequences in livestock worldwide [136–138]. Microscopy detection of these piroplasmids has little significance in the identification of various species [136, 139]. In contrast, molecular diagnostic technologies provide rapid, sensitive, and accurate species delimitation [140, 141]. Alternatively, to detect parasite-specific antibodies, serological diagnostic procedures such as the enzyme-linked immunosorbent assay (ELISA) and indirect immunofluorescence antibody test (IFAT) can be used [142, 143]. However, the presence of antibodies as a marker of earlier parasite exposure does not always correspond to the current infectious condition, as antibodies usually persist for variable periods of time [143]. Four methods have been used for the direct detection of *Babesia* piroplasm in the blood of infected buffaloes worldwide (Additional file 2: Fig. S1). In total, 25 datasets screened *Babesia* spp. DNA in the blood of 3593 buffaloes worldwide, and 460 cases were found infected, resulting in a pooled global prevalence of 12.0% (95% CI 9.2–14.7%). This prevalence was slightly higher than that estimated for 5517 buffaloes diagnosed using microscopy (i.e., stained blood smears) in 15 datasets; 654 animals were found positive, with a pooled prevalence of 10.0% (95% CI 7.0–13.0%). Asymptomatic carrier animals in which no parasitemia could be found by microscopic examination led to a high false-negative diagnosis rate [137, 144]. Contrastingly, polymerase chain reaction (PCR) enabled sensitive and precise detection of various *Theileria* and *Babesia* species in carrier animals [144–148].

Three datasets used the reverse line blot hybridization (RLB) assay to test 429 animals, yielding a lower prevalence of 5.9%. On the contrary, a comparatively high prevalence (18.2%) was detected when a single dataset used the loop-mediated isothermal amplification (LAMP) assay on 165 animals (Table 1). Sero-surveys were also conducted to detect *Babesia* in the blood of buffaloes using various tests (e.g., ELISA, IFAT, and latex agglutination test [LAT]). Enzyme-linked immunosorbent assay was used to test 1633 serum samples in 10 datasets, and 743 were found positive for antibodies against *Babesia* spp., with a pooled prevalence of 32.7% (95% CI 13.2–52.2%). The highest prevalence was estimated with serology, which is similar to a previous report [149]. This could be due to the nature of the disease, since naturally infected animals that survive the acute disease continue to be healthy carriers of the parasites with low parasitemia [143]. Therefore, the animals may be seropositive while evading detection by direct tests; in terms of sensitivity, microscopy has the lowest, followed by direct PCR, and nested PCR [142, 143]. Among molecular techniques, real-time PCR (qPCR) has shown better sensitivity and specificity [143]; however, the sensitivity depends on many factors, including target gene, origin of the sample, and level of parasitemia. Overall, the number of datasets detailing the prevalence of *Babesia* in buffaloes from Asian countries (*n* = 41) was four times that obtained from African (*n* = 10) or South American (*n* = 6) countries (Table 1). Not all datasets named the species of buffaloes tested; however, all datasets (*n* = 10) in Africa came from Egypt, and it can be assumed that all investigated buffaloes belonged to the Asian species (*B. bubalis*).

**Table 1:** Worldwide prevalence of Babesia infection in buffaloes, and variation in prevalence in relation to the detection method

Parameter	No. datasets	No. tested	No. positive	Pooled estimate % based on 95% CI	Heterogeneity *I*^2^%
Overall prevalence	57				
Blood smear	15	5517	654	10.0 (7.0–13.0)	98.11
PCR	25	3593	460	12.0 (9.2–14.7)	95.33%
RLB	3	429	46	5.9 (−1.8 to 13.7)	93.22%
LAMP	1	165	30	18.2 (12.3–24.1)	NA
ELISA	10	1633	743	32.7 (13.2–52.2)	98.93%
IFAT	2	163	46	29.5 (18.0–41.1)	53.07%
LAT	1	314	166	52.9 (47.3–58.4)	NA
Regional prevalence					
Africa	10				
Blood smear	2	93	15	40.1 (−26.1 to 106.3)	96.39%
PCR	4	313	18	4.1 (0.2–8.0)	74.42%
RLB	1	85	2	2.4 (−0.9 to 5.6)	NA
ELISA	3	421	180	37.7 (18.2–57.1)	94.05%
Asia	41				
Blood smear	13	5424	639	9.2 (6.1–12.3)	98.29%
PCR	17	2446	248	10.2 (7.4–13.0)	93.79%
RLB	2	344	44	7.8 (−5.2 to 20.8)	96.01%
LAMP	1	165	30	18.2 (12.3–24.1)	NA
ELISA	5	567	119	22.2 (11.1–33.2)	92.05%
IFAT	2	163	46	29.5 (18.0–41.1)	53.07%
LAT	1	314	166	52.9 (47.3–58.4)	NA
South America	6				
PCR	4	834	194	25.4 (5.6–45.3)	98.16%
ELISA	2	645	444	49.3 (3.5–63.5)	99.43%

*NA* not applicable

*Babesia bovis* and *B. bigemina* were the most frequently investigated and detected species in buffaloes based on either PCR sequencing or serological protocols, and *B. orientalis* were identified to a lesser extent (Additional file 1: Table S1). *Babesia bovis* and *B. bigemina* were the most common species in bovines worldwide. They are primarily found in tropical and subtropical regions of the world, including Australia, Africa, Asia, and the Americas, and are transmitted by the tick vectors *Rhipicephalus* (*Boophilus*) *microplus* and *R. annulatus*, and *R. decoloratus* for *B. bigemina* alone [149, 150]. Other species included *B. occultans* and *Babesia* sp. Mymensingh. Of note, *Babesia* sp. Mymensingh was recently named *Babesia naoakii* [151].

A large number of datasets were also found describing *Theileria* infections in buffaloes based either on direct detection methods (e.g., blood smear, PCR, RLB, LAMP) or serological assays for screening antibodies (e.g., ELISA and IFAT) (Additional file 3: Fig. S2). Like *Babesia*, the pooled global prevalence based on 3427 samples tested using PCR (24.6%; 95% CI 18.7–30.4%) was higher than that detected in 2447 samples tested microscopically (16.2%; 95% CI 10.6–21.9%) (Table 2). On the other hand, the estimated prevalence for ELISA-tested samples (*n* = 578) was 20.9% (95% CI 8.7–33.2%). Overall, the prevalence rates were much higher in Africa (12 datasets) than in Asia (34 datasets) for samples tested using either microscopy (66.7% and 13.4%, respectively) or PCR (42.0% and 20.7%, respectively); however, similar prevalence was estimated for both continents based on samples tested using the RLB (35.1 and 35.5%, respectively) (Table 2). Notably, the regional variation in the prevalence of *Theileria* spp. and *Babesia* spp. between Africa and Asia may be attributed to the difference in the number of datasets used and the species of buffaloes reared in Africa (*B. bubalis* and *S. caffer*) in comparison with the species reared in Asia (*B. bubalis*).

**Table 2:** Worldwide prevalence of *Theileria* infection in buffaloes, and variation in prevalence in relation to the detection method

Parameter	No. datasets	No. tested	No. positive	Pooled estimate % based on 95% CI	Heterogeneity *I*^2^ %
Overall prevalence	48				
Blood smear	11	2447	378	16.2 (10.6–21.9)	97.9%
PCR	27	3427	902	24.6 (18.7–30.4)	99.24%
RLB	4	681	265	35.6 (3.3–67.8)	99.41%
LAMP	2	254	73	28.7 (23.1–34.3)	0%
ELISA	2	578	109	20.9 (8.7–33.2)	90.83%
IFAT	2	127	99	85.9 (63.0–108.9)	94.54%
Regional prevalence					
Africa	12				
Blood smear	1	30	20	66.7 (49.8–83.5)	NA
PCR	7	456	202	42.0 (13.2–70.8)	99.53%
RLB	2	337	175	35.1 (−31.5 to 101.6)	99.79%
IFAT	2	127	99	85.9 (63.0–108.9)	94.54%
Asia	34				
Blood smear	10	2417	358	13.4 (7.9–18.9)	97.87%
PCR	18	2376	686	20.7 (12.6–28.8)	99.14%
RLB	2	344	90	35.5 (9.1–61.8)	90.61%
LAMP	2	254	73	28.7 (23.1–34.3)	0%
ELISA	2	578	109	20.9 (8.7–33.2)	90.83%
South America (PCR)	2	595	14	2.1 (−1.7 to 5.9)	90.44%

*NA* not applicable

Buffaloes have been found infected with different *Theileria* species, including *Theileria annulata*, *T. orientalis* complex (*orientalis/sergenti/buffeli*), and *T. parva* among the most frequently detected. *Theileria annulata* and *T. parva* (the causative agents of tropical or Mediterranean and East Coast fevers, respectively) are the most pathogenic species in bovines, whereas other species frequently cause asymptomatic infections in this host group [152]. Based on 18S ribosomal RNA sequences, phylogenetic analysis of *Theileria* genotypes reveals that the *T. buffeli* clade has the most genotypes and is found on all major continents, infecting cattle, African buffalo, water buffalo, and yak [137, 153, 154]. Members of this clade have previously been given a variety of species names, including *T. buffeli*, *T. orientalis*, and *T. sergenti*, and their taxonomy is debatable, but they have been proposed to constitute a single species known as *T. buffeli* [137, 153, 155] or *T. orientalis* complex in many other reports. *Theileria mutans*, *T. sinensis*, *T. velifera*, *T. lestoquardi*-like, *T. taurotragi*, *T*. sp. (buffalo), and *T. ovis* were also detected in naturally infected buffaloes.

### *Anaplasma* species

Anaplasmosis is an emerging infection that is gaining attention around the world because it affects animal body weight, causes abortions, reduces milk production, and leads to the death of animals [156]. Clinical disease is most common in cattle, although other ruminants, including water buffalo, could become persistently infected [157]. *Anaplasma marginale* was the most common tick-borne infection in buffaloes, and was considered the most widespread TBP globally in bovines, producing mild to severe hemolytic disease with significant economic loss [12, 158]. Forty-one datasets described *Anaplasma* infections in buffaloes, which represented the third most frequently tested TBPs in buffaloes worldwide after *Babesia* and *Theileria*, respectively (Additional file 4: Fig. S3). *Anaplasma* spp. were PCR-detected at a much higher rate than when microscopically detected (Table 3). Using blood smears, 886 samples in eight datasets were examined, and 118 were found positive resulting in pooled prevalence of 8.8% (95% CI 3.0–14.5%), whereas 5219 blood samples in 28 datasets were screened for *Anaplasma* DNA and 1330 were found positive with a pooled prevalence of 21.0% (95% CI 16.5–25.4%). Additionally, four datasets tested 868 serum samples using ELISA for the detection of *Anaplasma* spp., and 357 were found positive, giving rise to a prevalence of 27.8% (95% CI 2.4–53.2%). Moreover, RLB was used in a single study from Egypt for detection of *A. marginale*, with a prevalence rate of 42.4% (36/85), which is much higher than that of any other method.

**Table 3:** Worldwide prevalence of *Anaplasma* infection in buffaloes, and variation in prevalence in relation to the detection method

Parameter	No. datasets	No. tested	No. positive	Pooled estimate % based on 95% CI	Heterogeneity *I*^2^ %
Overall prevalence	41				
Blood smear	8	886	118	8.8 (3.0–14.5)	91.97%
PCR	28	5219	1330	21.0 (16.5–25.4)	98.79%
RLB	1	85	36	42.4 (31.8–52.9)	NA
ELISA	4	868	357	27.8 (2.4–53.2)	98.85%
Regional prevalence					
Africa	7				
Blood smear	1	85	2	2.4 (−0.9 to 5.6)	NA
PCR	5	1094	472	30.6 (3.1–58.2)	99.37%
RLB	1	85	36	42.4 (31.8–52.9)	NA
Asia	26				
Blood smear	7	801	116	9.8 (3.0–16.5)	92.37%
PCR	16	2638	730	22.2 (14.8–29.6)	98.57%
ELISA	3	368	112	20.6 (−5.5 to 46.8	98.08%
South America	6				
PCR	5	1339	82	6.9 (2.2–11.5)	94.96%
ELISA	1	500	245	49.0 (44.6–53.4)	NA
North America (PCR)	1	88	46	52.3 (41.8–62.7)	NA
Europe (PCR)	1	60	0	0.8 (−1.4 to 3.1)	NA

*NA* not applicable

In Africa, most studies relied on PCR for diagnosis of *Anaplasma* species, where 472 out of 1094 samples were found infected, with a pooled prevalence of 30.6% (95% CI 3.1–58.2%). The African buffalo was investigated in a single study from South Africa (Additional file 1: Table S1). Regarding Asia, PCR and ELISA were used in 16 and three datasets, respectively, with a nearly equal prevalence (22.2% and 20.6%, respectively), while lower prevalence was estimated based on microscopy (9.8%). *Anaplasma marginale*, *A. centrale*, *A. platys*, *A. platys*-like, and “*Candidatus* Anaplasma boleense” were recovered from infected buffaloes, with *A. marginale* being the most frequently investigated and detected species. *Anaplasma platys* and *A. platys*-like were detected in infected water buffaloes from Egypt, Thailand, and Malaysia [31, 34, 80, 107]. *Anaplasma centrale* was found infecting water buffaloes from Pakistan [82], and African buffaloes in South Africa and Uganda [37, 42]. Additionally, “*Candidatus* A. boleense” was recorded in a single study in water buffaloes from Malaysia [80]. However, *A. phagocytophilum* has a broad host range including humans [159]; only one study investigated the parasite in 60 water buffaloes in Hungary, and no infection was detected by qPCR [120].

### *Coxiella burnetii*

The intracellular Gram-negative bacterium *Coxiella burnetii*, which can be excreted in tick feces and saliva, is the cause of Q fever, a zoonotic infection that is globally transmitted [160, 161]. Because of the prevalence of this bacterium in ticks from different bioclimatic zones and socioeconomic contexts, it is clear that ticks play a significant role in the epidemiology of Q fever [162, 163]. *Coxiella burnetii* infection leads mainly to reproductive disorders including abortions, premature birth, stillbirth, and poor calf deliveries, which have a negative economic impact on livestock [164–166]. Infection with *C. burnetii* in humans is typically asymptomatic or appears like the flu and can result in acute and chronic fever disease and pneumonia. It is mostly transmitted to humans by aerosolized dry, contaminated soil or animal products [160, 162, 166, 167].

*Coxiella burnetii* infections were tested in sera of buffaloes in six datasets worldwide using ELISA (five datasets) and IFAT (one set). A total of 987 sera were examined using ELISA, and 63 harbored the antibodies against *C. burnetii*, resulting in a pooled prevalence of 5.3% (95% CI 1.4–9.2%) (Table 4). The prevalence of *Coxiella burnetii* in buffaloes from Africa was higher (7.5%) than that in Asia (3.8%) based on ELISA. A higher number of samples were examined using PCR (1360 samples) including buffaloes’ blood, milk, vaginal discharges, vaginal and preputial swabs, placental cotyledons, and aborted fetuses (Additional file 1: Table S1), which were used for estimating the pooled prevalence (4.5%; 95% CI 1.9–7.1%). The detection of *Coxiella burnetii* in buffaloes’ sera along with the detection in raw milk [68, 69, 76] suggests that they can play a role in the transmission of Q fever to humans, but more extensive prevalence studies need to be carried out to define the role of buffaloes as reservoirs for this pathogen. Furthermore, the role of *C. burnetii* as an abortive agent in buffaloes has been suggested [121].

**Table 4:** Worldwide prevalence of *C. burnetii* infection in buffaloes, and variation in prevalence in relation to the detection method

Parameter	No. datasets	No. tested	No. positive	Pooled estimate % based on 95% CI	Heterogeneity *I*^2^ %
Overall prevalence	15				
PCR	9	1360	106	4.5 (1.9–7.1)	92.71%
ELISA	5	987	63	5.3 (1.4–9.2)	91.54%
IFAT	1	156	7	4.5 (1.2–7.7)	NA
Regional prevalence					
Africa	4				
PCR	2	52	0	1.9 (−1.7 to 5.4)	0%
ELISA	2	457	40	7.5 (0.4–14.6)	89.14%
Asia	10				
PCR	6	1144	92	4.6 (1.5–7.6)	94.92%
ELISA	3	530	23	3.8 (−0.3 to 7.9)	88.84%
IFAT	1	156	7	4.5 (1.2–7.7)	NA
Europe (PCR)	1	164	14	8.5 (4.3–12.8)	NA

*NA* not applicable

### Miscellaneous pathogens

Isolated surveys have detected a few potentially zoonotic TBPs infecting buffaloes worldwide (Table 5). Notably, 5617 sera from three datasets in Brazil were examined for *Borrelia burgdorferi* and 4157 were found to harbor antibodies, resulting in a pooled prevalence of 68.2% (95% CI 53.2–83.1%). PCR was used to screen *B. burgdorferi* sensu lato in eight buffaloes in the Czech Republic, and a single case was found infected (12.5%) in contrast to the examined 26 buffaloes in Egypt for *Borrelia theileri*, where no cases were found infected (Additional file 1: Table S1). *Borrelia burgdorferi* is a tick-borne obligatory parasite with a natural reservoir of a range of mammals, although, infection of these natural hosts does not result in disease; nonetheless, infection of humans can result in Lyme disease [168–170].

**Table 5:** Worldwide prevalence of miscellaneous tick-borne pathogens of buffaloes, and variation in prevalence in relation to the detection method

Pathogen	Parameter	No. datasets	No. tested	No. positive	Pooled estimate % based on 95% CI	Heterogeneity *I*^2^ %
*Borrelia* spp.	Overall prevalence	5				
	PCR	2	34	1	2.4 (−2.6 to 7.3)	0%
	iELISA	3	5617	4157	68.2 (53.2–83.1)	98.67%
	Regional prevalence					
	Africa (PCR)	1	26	0	1.9 (−3.2 to 6.9)	NA
	South America (iELISA)	3	5617	4157	68.2 (53.2–83.1)	98.67%
	Europe (PCR)	1	8	1	12.5 (−10.4 to 35.4)	NA
*Bartonella* spp.	Overall prevalence	5				
	PCR	3	208	3	1.5 (−1.9 to 5.0)	42.93%
	Culture	1	103	7	6.8 (1.9–11.7)	NA
	IFAT	1	156	25	16.0 (10.3–21.8)	NA
	Regional prevalence					
	Africa (PCR)	2	52	3	5.0 (−3.9 to 13.9)	50.99%
	Asia	3				
	PCR	1	156	0	0.3 (−0.6 to 1.2)	NA
	Culture	1	103	7	6.8 (1.9–11.7)	NA
	IFAT	1	156	25	16.0 (10.3–21.8)	NA
*Ehrlichia* spp.	Overall prevalence (PCR)	4	328	13	3.1 (−0.3 to 6.5)	73.39%
	Regional prevalence					
	Africa	1	95	3	3.2 (−0.4 to 6.7)	NA
	Asia	3	233	10	3.5 (−1.5 to 8.5)	79.77%
CCHFV	Overall prevalence (ELISA)	4	880	145	16.0 (9.9–22.1)	99.48%
	Regional prevalence					
	Africa	3	608	145	23.8 (12.4–35.3)	99.65%
	Asia	1	272	0	0.2 (−0.3 to 0.7)	NA
Rickettsiae	Overall prevalence (PCR)	4	220	0	0.7 (−0.4 to 1.7)	0%

*iELISA* indirect ELISA, *NA* not applicable

*Bartonella* species are arthropod-borne Gram-negative bacteria that infect erythrocytes, endothelial cells, and macrophages, frequently resulting in chronic blood-borne infections [171]. They are the causative agents of multiple human diseases, and their main vectors include fleas, keds, lice, sand flies, bed bugs, biting flies, and ticks [171–173]. Ticks were previously postulated but not confirmed as a vector for *Bartonella* transmission; nevertheless, there is increasing evidence of transovarial and transstadial transmission of bartonellae in ixodid ticks [174–176]. A pooled prevalence of 1.5% (95% CI −1.9 to 5.0%) was estimated for *Bartonella* spp. in buffaloes from three datasets based on PCR. Other methods were used based on traditional culture and IFAT for the detection of *Bartonella* spp. and resulted in higher prevalence (6.8% and 16%, respectively) from one dataset for each method. *Bartonella bovis* was identified in water buffaloes from Thailand based on multi-locus sequence typing in a single study [105]. *Bartonella henselae*, *B. vinsonii* subsp. *berkhoffii*, and *B. tamiae* antibodies were found in buffaloes from Thailand using indirect IFAT; however, PCR results were negative [109].

Much lower prevalence of 3.1% (95% CI −0.3 to 6.5%) was estimated for 328 buffaloes tested for ehrlichiosis with PCR from four datasets, which constitutes a potentially fatal emerging zoonosis of global veterinary concern [177]. *Ehrlichia ruminantium* was the species identified from African buffaloes in Namibia [40]. In South Africa, a buffalo calf (*S. caffer*) was suspected of having died as a result of heartwater (*E. ruminantium*) [178]. However, small samples were used for the detection of *Rickettsia* species; 220 buffaloes from three continents, namely Africa (Egypt), Asia (Philippines), and Europe (Hungary), were found free from infection based on PCR [33, 34, 98, 120].

Crimean-Congo hemorrhagic fever (CCHF) is a viral infection spread by ticks. Humans are the only known hosts that develop sickness following CCHFV infection, owing to the virus's asymptomatic presence in animal reservoirs, which allows it to circulate undetected [179]. Humans can become infected with CCHFV through tick bites or contact with contaminated animal tissues during and immediately after slaughter [180, 181]. Notably, humans who had high CCHF seropositivity had a history of animal contact, animal husbandry, farming, and tick bites [182]. Buffaloes can act as silent reservoirs of CCHFV; the sera of 880 buffaloes were examined using ELISA from four datasets and 145 were found infected, with a pooled prevalence of 16.0% (95% CI 9.9–22.1%).

## Conclusions

The present article provides the first meta-analysis of the published data on TBPs in buffaloes. The analyses conducted have some limitations sometimes due to the limited number of available studies (e.g., rickettsioses) as well as variability in the relevance of the reported data and diagnostic methods used. Nonetheless, the close interaction of varied animal species, the availability of mixed animal shelters, and unregulated animal movements in many African and Asian countries all increase the risk of a pathogen crossing the species barrier. This is evident in many species and genotypes of *Babesia*, *Theileria*, and *Anaplasma* that are circulating between buffaloes and cattle worldwide. Consequently, detection of the pathogenic species is considered critical not only for buffaloes but also for cattle raised in the same areas, with economic implications. There is evidence of high species diversity of *Babesia*, *Theileria*, and *Anaplasma* infecting buffaloes, which suggests susceptibility to a wide range of TBPs, and adequate control measures should be applied to prevent their circulation.

## Supplementary Information

**Additional file 1: Table S1.** Study characteristics of tick-borne pathogen surveys in buffaloes worldwide.

**Additional file 2: Figure S1.** Forest plot diagrams for random effects in the meta-analysis of the prevalence of *Babesia* spp. infections in buffaloes worldwide. The middle point of each line indicates the prevalence, while the length of the line is the 95% confidence interval for each study. Diamonds refers to the prevalence in accordance with detection methods.

**Additional file 3: Figure S2.** Forest plot diagrams for random effects in the meta-analysis of the prevalence of *Theileria *spp. infections in buffaloes worldwide. The middle point of each line indicates the prevalence, while the length of the line is the 95% confidence interval of each study. Diamonds refers to the prevalence in accordance with detection methods.

**Additional file 4: Figure S3.** Forest plot diagrams for random effects in the meta-analysis of the prevalence of *Anaplasma *spp. infections in buffaloes worldwide. The middle point of each line indicates the prevalence, while the length of the line is the 95% confidence interval of each study. Diamonds refers to the prevalence in accordance with detection methods.

## Data Availability

All data generated or analysed during this study are included in this published article and its supplementary information files.
