# Supplementary material for: Global prevalence and species diversity of tick-borne pathogens in buffaloes worldwide: a systematic review and meta-analysis
Source: Parasit Vectors. 2023 Mar 30;16:115. doi: 10.1186/s13071-023-05727-y (PMC10061416; doi:10.1186/s13071-023-05727-y)
Supplement: Supplementary file 1 — Additional file 1: Table S1. Study characteristics of tick-borne pathogen surveys in buffaloes worldwide. [file 13071_2023_5727_MOESM1_ESM.docx]

**Additional file Information Table S1**: Study characteristics of tick-borne pathogens surveys in buffaloes worldwide.

| **Region** | **Buffalo species** | **Country** | **Detection method#** | **Study Year** | **Sample size** | **No. positive** | **Prevalence (%)** | **Marker** | **Species** | **Reference** |
| --- | --- | --- | --- | --- | --- | --- | --- | --- | --- | --- |
| **Africa** | *Syncerus caffer* | Botswana | RLB  IFAT  qPCR | N.S. | 120  108  119 | 72  44  36  24  28  7  80  96 | 60  37  30  20  23  6  74  81 | 18S rRNA  16S rRNA  -  - | *Theileria parva*  *T. mutans*  *Anaplasma marginale sub central*  *A. marginale*  *Babesia occultans*  *Ehrlichia ruminantium*  *T. parva*  *T. parva* | Eygelaar et al. [20] |
|  | *Bubalus bubalis* | Egypt | Blood smear | N.S. | 30 | 20 | 66.6 | N.S. | *Theileria annulata* | Osman and Al-Gaabary [21] |
|  | *Bubalus bubalis* | Egypt | ELISA | 2004- 2005 | 264 | 1 | 0.38 | - | CCHFV | Mohamed et al. [22] |
|  | *Bubalus bubalis* | Egypt | n PCR/ seq.  ELISA | 2011 | 96 | 1  10  4  5  15  11 | 1.04  10.42  4.17  5.21  15.63  11.46 | rap-1  SBP-4 | Mixed  *Babesia bigemina*  *B. bovis*  Mixed  *B. bigemina*  *B. bovis* | Ibrahim et al. [23] |
|  | *Bubalus bubalis* | Egypt | Blood smear | 2008 | 12 | 9 | 75 | - | *Babesia bovis* | Mahmmod [24] |
|  | N.S. | Egypt | ELISA | 2009 | 153 | 6  0 | 4  0 | - | *Coxiella burnetii*  CCHFV | Horton et al. [25] |
|  | N.S. | Egypt | n PCR/ seq. | 2013 | 50 | 1  1 | 2  2 | rap-1  MPSP | *Babesia bovis*  *Theileria orientalis* | Elsify et al. [26] |
|  | N.S. | Egypt | Blood smear  cELISA  sn PCR/ seq.  n PCR | N.S. | 81 | 6  5  18  18  3  0 | 7.4  6.2  22.2  22.2  3.7  0 | rra  rap-1c | *Babesia* spp.  Mixed  *B bovis*  *B. bigemina*  *B bovis*  *B. bigemina* | Mahmoud et al. [27] |
|  | *Bubalus bubalis* | Egypt | n PCR (placental cotyledons and vaginal discharges | N.S. | 26 | 0 | 0 | IS1111 | *Coxiella burnetii* | Abdel-Moein and Hamza [28] |
|  | *Bubalus bubalis* | Egypt | PCR | N.S. | 150 | 104 | 69.3 | msp1α | *Anapalsma marginale* | ELHariri et al. [29] |
|  | N.S. | Egypt | ELISA | 2015- 2016 | 304 | 34 | 11.2 | - | *Coxiella burnetii* | Klemmer et al. [30] |
|  | N.S. | Egypt | Blood smear  qPCR  RLB  RLB  RLB  RLB | 2018 | 85 | 2  25  36  1  2  4 | 2.4  29.4  42.4  1.18  2.35  4.71 | -  msp1β  groEL  18S rRNA  18S rRNA  16S rRNA | *Anapalsma marginale*  *A. marginale*  *A. marginale*  *Theileria annulata*  *Babesia bigemina*  *A. platys* | AL-Hosary et al. [31] |
|  | *Bubalus bubalis* | Egypt | PCR/seq. | 2017 | 86 | 1  0  1  0 | 1.2  0.0  1.2  0.0 | SBP4  Rap1a  groEL  18S rRNA | *Babesia bovis*  *B. bigemina*  *Anapalsma marginale*  *Theileria* sp. | Tumwebazeet al. [32] |
|  | *Bubalus bubalis* | Egypt | PCR/seq. | 2019-2020 | 26 | 3  0  0 | 11.54  0.0  0.0 | ITS  16S rRNA  gltA | *Bartonella* sp.  *Borrelia theileri*  *Rickettsiae* | Abdullah et al. [33] |
|  | N.S. | Egypt | qPCR  cPCR  qPCR  cPCR  qPCR  qPCR  qPCR | 2016 - 2018 | 26 | 2  2  2  2  0  0  0 | 7.7  7.7  7.7  7.7  0.0  0.0  0.0 | 5.8S rRNA  18S rRNA  23S rRNA  Ana-rpoB  gltA  IS1111  ITS3 | *Piroplasmida*  *Theileria ovis*  *Anaplasmataceae*  *A. platys-like*  *Rickettsia sp.*  *Coxiella burnettii*  *Bartonella sp* | Abdullah et al. [34] |
|  | *Bubalus bubalis* | Egypt | ELISA | 2017- 2018 | 244 | 37  87  78 | 15.1  35.66  31.97 | - | Mixed  *Babesia bigemina*  *B. bovis* | Ibrahim et al. [35] |
|  | *Syncerus caffer* | South Africa | RLB | N.S. | 252 | 66  17  14  26  8  1  108  38  68  79  62  59 | 26.2  6.8  5.6  10.3  3.2  0.4  42.9  15.1  30.0  31.3  24.6  23.4 | 18S rRNA | Single *Theileria*:  *T. buffeli*  *T. mutans*  *T. parva*  *Theileria* sp. (buffalo)  *T. velifera*  Mixed *Theileria*:  *T. buffeli*  *T. mutans*  *T. parva*  *Theileria* sp. (buffalo)  *T. velifera* | Chaisi et al. [36] |
|  | *Syncerus caffer* | South Africa | n PCR/seq. | 2014- 2016 | 747 | 129  98  113 | 17.3  13.1  15.1 | msp1β  groEL | *Anapalsma marginale*  *A. centrale*  Both spp. | Sisson et al. [37] |
|  | *Syncerus caffer* | South Africa | qPCR  IFAT | 2009- 2013 | 19 | 19  19 | 100  100 | N.S. | *Theileria parva* | Latif et al. [38] |
|  | *Syncerus caffer* | Kenya | ELISA | 2008- 2018 | 191 | 144 | 75.3 | - | CCHFV | Obanda et al. [39] |
|  | *Syncerus caffer* | Namibia | QPCR  PCR | 2009 | 95 | 54  3 | 57  3 | 18S rRNA  map1 | *Theileria parva*  *Ehrlichia ruminantium* | [Pascucci](https://pubmed.ncbi.nlm.nih.gov/?term=Pascucci+I&cauthor_id=22204058) et al. [40] |
|  | N.S. | Tanzania | n PCR | N.S. | 61 | 30 | 49 | p104 | *Theileria parva* | Rukambile et al. [41] |
|  | *Syncerus caffer* | Uganda | RLB  n PCR (MS 16)  RLB  RLB  RLB  RLB  RLB  RLB  RLB | N.S. | 83 | 34  36  81  0  78  21  40  53  48 | 41  43  97.5  0  93.9  25.3  48.1  63.8  57.8 | N.S. | *Theileria parva*  *T. parva*  *T. mutans*  *T. taurotragi*  *T. velifera*  *T. buffeli*  *T. spa* (buffalo)  *Anaplasma central*  *A. marginale* | Oura et al. [42]* |
|  |  |  |  |  |  |  |  |  |  |  |
| **Asia** | *Bubalus bubalis* | China | LAT | N.S. | 230  84 | 165  1 | 71.7^1^  1.19^2^ | - | *Babesia orientalis* | Baoan et al. [43] |
|  | N.S. | China | Blood smear  sn PCR | 2005 | 121 | 5  24 | 4.1  19.8 | 18S rRNA | *Babesia orientalis* | Liu et al. [44] |
|  | *Bubalus bubalis* | China | LAMP  sn PCR | N.S. | 165 | 30  13 | 18.2  7.9 | 18S rRNA | *Babesia orientalis* | He et al. [45] |
|  | N.S. | China | Blood smear  sn PCR  rBoP29-ELISA | N.S. | 132 | 37  44  39 | 28  33.3  29.5 | rBoP29 | *Babesia orientalis* | Zhou et al. [46] |
|  | *Bubalus bubalis* | China | i ELISA  PCR  Blood smear | N.S. | 178 | 49  40  23 | 27.5  22.5  12.9 | 18S rRNA | *Theileria sergenti* | Wang et al. [47] |
|  | *Bubalus bubalis* | China | LAMP  PCR | N.S. | 154 | 46  38 | 30  24.7 | p33 | *Theileria sergenti* | Wang et al. [48] |
|  | *Bubalus bubalis* | China | RLB/ seq. | N.S. | 304 | 58  27  3  2  10  1  1 | 19.1  8.9  1  0.7  3.3  0.3  0.3 | 18S rRNA | Single infections:  *Theileria buffeli*  *Babesia orientalis*  *B. bovis*  *B. bigemina*  Mixed infections:  *T. buffeli+B. orientalis*  *T. buffeli+B. bovis*  *T. buffeli+B. bovis+B. orientalis* | He et al. [49] |
|  | *Bubalus bubalis* | China | pan-Theileria FRET-qPCR | 2007- 2013 | 29 | 0 | 0 | 18S rRNA | *Theileria* spp. | Yang et al. [50] |
|  | *Bubalus bubalis* | China | FRET-qPCR | 2007- 2013 | 29  29 | 2  0 | 6.9  0 | 16SrRNA | *Anaplasma* spp.  *Ehrlichia* spp. | Qiu et al. [51] |
|  | *Bubalus bubalis* | China | PCR / seq. | 2015- 2020 | 85 | 0 | 0 | 18S rRNA | *Babesia* spp. | Zeng et al. [52] |
|  | N.S. | India | SELISA  IFAT  Blood smear | N.S. | 120 | 36  30  0 | 30  25  0 | - | *Babesia bigemina* | Singh et al. [53] |
|  | N.S. | India | PCR  Blood smear | 2011 | 22 | 4  0 | 18.1  0 | mspIβ | *Anaplasma marginale*  *Anaplasma* spp. | Sharma et al. [54] |
|  | N.S. | India | Duplex PCR  Blood smear | 2011 | 25 | 2  1 | 8  4 | SSU rRNA | *Babesia bigemina* | Sharma et al. [55] |
|  | N.S. | India | Trans-PCR/ seq. | N.S. | 60 | 5 | 8.3 | IS1111 | *Coxiella burnetii* | Das et al. [56] |
|  | N.S. | India | qPCR | N.S. | 25 | 8 | 32 | Tams 1 | *Theileria annulata* | Kundave et al. [57] |
|  | N.S. | India | ELISA | N.S. | 69 | 4  2 | 5.8  2.9 | - | *Babesia bigemina*  *Anaplasma marginale* | Filia et al. [58] |
|  | N.S. | India | Blood smear | 2011- 2014 | 226 | 46  16  12 | 20.35  7.07  5.30 | - | *Babesia bigemina*  *Anaplasma marginale*  *Theileria annulata* | Maharana et al. [59] |
|  | N.S. | India | Blood smear  i ELISA  PCR / seq. | 2011- 2013 | 55 | 3  10  14 | 5.45  18.18  25.45 | mspI𝛽 | *Anaplasma marginale* | Sharma et al. [60] |
|  | N. S. | India | i ELISA  Blood smear | N.S. | 203 | 23  2 | 11.3  0.98 | - | *Babesia bigemina* | Kaur et al. [61] |
|  | N.S. | India | Blood smear | 2012- 2013 | 85 | 11  4  2 | 12.9  4.7  2.3 | - | *Theileria* spp.  *Babesia bigemina*  *Anaplasma marginale* | Krishna murthy et al. [62] |
|  | N.S. | India | Blood smear  PCR/ sequencing | 2011 | 76  76 | 6  1 | 7.8  1.31 | 18S rRNA | *Babesia* spp.  *B. bigemina* | Sharma et al. [63] |
|  | N.S. | India | Blood smear | 2017 | 382 | 117  0 | 30.6  0 | - | *Theileria annulata*  *Babesia bigemina* | Khorajiya et al. [64] |
|  | N.S. | India | Blood smear  PCR | N.S. | 209 | 26  50 | 12.44  23.92 | SSU rDNA | *Babesia bigemina* | Maharana et al. [65] |
|  | *Bubalus bubalis* | India | PCR/ seq.  Blood smear | N.S. | 60 | 11  4 | 18.3  6.7 | msp5  - | *Anaplasma marginale* | Kumar et al. [66] |
|  | N.S. | India | Blood smear  PCR | N.S. | 79 | 7  18 | 8.8  22.7 | Tams 1 | *Theileria annulata* | Patil and Satbige [67] |
|  | N.S. | India | ELISA  Trans-PCR (Milk, vaginal and preputial swabs) | 2017-2018 | 232 | 9  2 | 3.9  0.9 | IS1111 | *Coxiella burnetii* | Keshavamurthy et al. [68]- [69]* |
|  | N.S. | India | trans-PCR  iELISA | N.S. | 168 | 1  14 | 0.6  8.3 | IS1111, com1 | *Coxiella burnetii* | Dhaka et al. [70] |
|  | N.S. | India | PCR  Blood smear | N.S. | 487 | 5  3 | 1.02  0.61 | SpeI-AvaI | *Babesia bigemina* | Kaur et al. [71] |
|  | N.S. | India | C ELISA | N.S. | 244 | 100 | 40.9 | - | *Anaplasma* spp. | Sarangi et al. [72] |
|  | N.S. | India | LAMP  PCR/ seq.  Blood smear | N.S. | 100 | 27  23  16 | 27  23  16 | ITS  Tams1  - | *Theileria annulata* | Kumar et al. [73] |
|  | *Bubalus bubalis* | India | PCR/seq. | 2020- 2021 | 114 | 32 | 28.07 | msp5 | *Anaplasma marginale* | Ntesang et al. [74] |
|  | N.S. | Iran | Blood smear  sn PCR | 2014 | 291 | 4  4  2  2 | 1.4  1.4  0.7  0.7 | 18S rRNA | *Theileria annulata*  *T. annulata*  *T. orientalis.*  Mixed *Theileria* spp. | Narimani et al. [75] |
|  | *Bubalus bubalis* | Iran | n PCR (Milk samples) | 2017 | 420 | 81 | 19.3 | IS1111 | *Coxiella burnetii* | Khademi et al. [76] |
|  | *Bubalus bubalis* | Iran | Blood smear  PCR | N.S. | 103 | 16  32 | 15.5  31.1 | groEL | *Anaplasma*-like  *A. marginale* | Nikvand et al. [77] |
|  | N.S. | Iraq | Blood smear  PCR/seq.  PCR/seq. | 2017 | 94 | 43  36  7 | 45.7  38.30  7.45 | SSU rRNA  SSU rRNA | *Babesia* spp.  *B. bovis*  *B. bigemina* | Ateaa and Alkhaled [78] |
|  | N.S. | Laos | i ELISA | 2013- 2015 | 130 | 0 | 0 | - | *Coxiella burnetii* | Douangngeun et al. [79] |
|  | *Bubalus bubalis* | Malaysia | n PCR/seq. | 2013 | 55 | 12 | 21.8 | 16S rRNA | ***Anaplasmataceae***  “*Candidatus* Anaplasma boleense”  *A. platys / A. phagocytophilum*  *A. bovis* | Koh et al. [80] |
|  | *Bubalus bubalis* | Myanmar | n PCR/ seq. | 2016 | 17 | 2 | 11.8 | 18S rRNA | *Theileria velifera*-like | Bawm et al. [81] |
|  | *Bubalus bubalis* | Pakistan | Blood smear | 2005 | 250 | 75  34  21  20 | 30  13.6  8.4  8 | - | *Anaplasma* spp.  *A. maginale*  *A. centrale*  Mixed both | Rajput et al. [82] |
|  | N.S. | Pakistan | Blood smear  n-PCR | 2007 | 336 | 134  179 | 39.9  53.3 | -  ssu-rRNA | *Theileria* sp.  *T. annulata.* | Durrani et al. [83] |
|  | N.S. | Pakistan | RLP/ seq. | N.S. | 40 | 20  0 | 50  0 | 18S rRNA | *Theileria annulata*  *Babesia* sp. | Khan et al. [84] |
|  | N.S. | Pakistan | PCR | 2010 | 39 | 9 | 23 | SSU rRNA | *Babesia bovis* | Zulfiqar et al. [85] |
|  | *Bubalus bubalis* | Pakistan | PCR/seq.  RFLP-PCR | 2011 | 281 | 116 | 41 | 16S rRNA  BssNa1 | *Anaplasma* sp.  *A. marginale* | Ashraf et al. [86] |
|  | N.S. | Pakistan | PCR/seq. | 2015 | 421 | 34 | 8.07 | SBP-4 | *Babesia bovis* | Farooqi et al. [87] |
|  | *Bubalus bubalis* | Pakistan | MT-PCR/ seq. | 2015 | 49 | 3 | 6.1 | MPSP | *Theileria orientalis* | Gebrekidan et al. [88] |
|  | N.S. | Pakistan | PCR/seq. | N.S. | 421 | 62 | 14.7 | 16S rRNA | *Anaplasma marginale* | Farooqi et al. [89] |
|  | N.S. | Pakistan | Blood smear  i ELISA  QPCR | N.S. | 400 | 32  60  71 | 8  15  17.7 | - | *Theileria parva* | Rafiullah et al. [90] |
|  | *Bubalus bubalis* | Pakistan | PCR/seq. | 2017 | 154 | 70  59  1  14  9  13 | 45.5  38.3  0.6  9.09  5.8  8.4 | 18S rRNA | ***Piroplasmida***  *Theileria annulata*  *T. lestoquardi-like*  *Babesia bovis*  *B. bigemina*  Mixed piroplasms | Ghafar et al. [91]* |
|  | N.S. | Pakistan | Blood smear | N.S. | 3264 | 466 | 14.28 | - | *Babesia* spp. | Siddique et al. [92] |
|  | N.S. | Pakistan | PCR/seq. | N.S. | 96 | 10 | 10.4 | sodb | *Ehrlichia* spp. | Basit et al. [93] |
|  | N.S. | Pakistan | Blood smear | 2017- 2018 | 340 | 2 | 0.59 | - | *Theileria* spp. | Mohsin et al. [94] |
|  | *Bubalus bubalis* | Philippines | n PCR  PCR | N.S. | 272 | 28  12 | 10.3  4.4 | msp1 β  AvaI–SpeI | *Anaplasma marginale*  *Babesia bigemina* | Mingala et al. [95] |
|  | *Bubalus bubalis* | Philippines | n PCR/ seq. | N.S. | 65 | 0  0 | 0  0 | SBP2  rap-1 | *Babesia bovis*  *B. bigemina* | Herrera et al. [96] |
|  | *Bubalus bubalis* | Philippines | PCR/seq. | 2016- 2017 | 100 | 46  29  21  3  1  0  0  0 | 46  29  21  3  1  0  0  0 | -  groEL  SBP-2  rap-1a  -  Tams 1  MPSP  ama-1 | **TBPs**  *Anaplasma marginale*  *Babesia bovis*  *B. bigemina*  Mixed *Babesia*  *Theileria annulata*  *T. orientalis,*  *B. ovata* | Galon et al. [97] |
|  | *Bubalus bubalis* | Philippines | n PCR/ seq. | 2016- 2019 | 108 | 0  3 | 0  2.8 | gltA  com1 | *Rickettsiae*  *Coxiella burnetii* | Galay et al. [98] |
|  | *Bubalus bubalis* | Philippines | PCR | 2018 | 114 | 0  0  0  0 | 0  0  0  0 | groEL  SBP-2  RAP-1a  18S rRNA | *Anaplasma marginale*  *Babesia bovis*  *B. bigemina,*  *Theileria* spp. | Galon et al. [99] |
|  | *Bubalus bubalis* | Philippines | nPCR/seq.  PCR/seq. | 2016- 2019 | 108 | 13  1  0 | 12  0.9  0 | groEL  msp5  dsbA | ***Anaplasmataceae***  *A. marginale*  *Ehrlichia* sp. | Galay et al. [100] |
|  | *Bubalus bubalis* | Sri Lanka | n PCR/ seq.  PCR/seq.  PCR/seq. | 2013 | 320 | 6  5  264 | 1.9  1.6  82.5 | rap-1  ama-1  MPSP | *Babesia* *bovis*  *B*. *bigemina*  *Theileria* *orientalis* | Sivakumar et al. [101] |
|  | N.S. | Sri Lanka | PCR/seq. | N.S. | 327 | 188 | 57.5 | msp5 | *Anaplasma* *marginale* | Zhyldyz et al. [102] |
|  | N.S. | Sri Lanka | PCR/seq. | 2013- 2018 | 327 | 4 | 1.2 | ama-1 | *Babesia* sp. Mymensingh | Sivakumar et al. [103]* |
|  | *Bubalus bubalis* | Thailand | n PCR/seq  ELISA  IFAT | 2010 | 305 | 34  11  45  18  51  17 | 11.2  3.6  14.7  5.9  16.8  5.6 | SBP-4  rap-1a | *Babesia bovis*  *B.bigemina*  *B. bovis*  *B.bigemina*  *B. bovis*  *B.bigemina* | Terkawi et al. [104]* |
|  | *Bubalus bubalis* | Thailand | Culturing  PCR/seq. | N.S. | 103  - | 7  7 | 6.8  - | -  gltA | ***Bartonella* spp.**  *B. bovis* | Bai et al. [105] |
|  | *Bubalus bubalis* | Thailand | PCR | 2017- 2018 | 121 | 0 | 0 | msp4 | *Anaplasma marginale* | Junsiri et al. [106] |
|  | *Bubalus bubalis* | Thailand | PCR/ seq. | 2015- 2018 | 456  40  40 | 187  37  3 | 41  92.5  7.5 | 16S rRNA | ***Anaplasmataceae***  *A. marginale*  *A. platys* | Nguyen et al. [107] |
|  | *Bubalus bubalis* | Thailand | PCR/ seq. | 2015- 2018 | 456  - | 116  12 | 25.4 | 18S rRNA | **Piroplasmida**  *Theileria orientalis* | Nguyen et al. [108]* |
|  | *Bubalus bubalis* | Thailand | Indirect IFA  PCR | 2021 | 156  25  25  25 | 25  14  5  6  0 | 16  56  20  24  0 | -  rpoB | ***Bartonella* spp.**  *B. henselae*  *B. vinsonii* subspp. *berkhoffii*  *B. tamiae*  ***Bartonella* spp.** | Boonmar et al. [109] |
|  | *Bubalus bubalis* | Thailand | PCR  Indirect IFA | 2021 | 156  156 | 0  7 | 0  4.49 | cox | *Coxiella burnetii* | Kidsin et al. [110] |
|  | N.S. | Thailand -  Cambodia border | PCR | 2020 | 49 | 3 | 6.1 | 18S rRNA | *Theileria spp.* | Kaewhom and  Srikijkasemwat [111] |
|  | *Bubalus bubalis* | Turkey | ELISA | 2017- 2018 | 272 | 0 | 0 | - | CCHFV | Okur-Gumusova et al. [112] |
|  | *Bubalus bubalis* | Vietnam | PCR/ seq. | 2010 | 43 | 11 | 25.6 | MPSP | *Theileria orientalis* | Khukhuu et al. [113] |
|  | *Bubalus bubalis* | Vietnam | n PCR/ seq.  Single PCR/ seq. | 2010 | 43 | 4  0  0 | 9.3  0  0 | SBP-2  ama –1  MPSA | *Babesia bovis*  *B. bigemina*  *Theileria annulata* | Sivakumar et al. [114] |
|  | *Bubalus bubalis* | Vietnam | n PCR  ELISA  IFAT | 2010 | 43 | 10  0  0  16  4  3  12  8  4 | 23.3  0  0  37.2  9.3  7  27.9  18.6  9.3 | SBP-2  rap-1a | *Babesia bovis*  *B. bigemina*  Mixed  *B. bovis*  *B. bigemina*  Mixed  *B. bovis*  *B. bigemina*  Mixed | Li et al. [115] |
|  | *Bubalus bubalis* | Vietnam | n PCR/seq. | 2013 | 49 | 16 | 32.7 | rap-1 | *Babesia bovis* | Yokoyama et al. [116]* |
|  | *Bubalus bubalis* | Vietnam | PCR | 2013 | 49 | 16  2  0  1  22 | 32.7  4.1  0  2.04  44.9 | ama –1  MPSP  ama-1  -  MPSP | *Babesia bovis*  *B. bigemina*  *B. ovata*  Mixed *Babesia*  *Theileria orientalis* | Weerasooriya et al. [117] |
|  | N.S. | Vietnam | PCR/seq. | 2010- 2013 | 92 | 10 | 10.9 | ama-1 | *Babesia* sp. Mymensingh | Sivakumar et al. [103]* |
|  |  |  |  |  |  |  |  |  |  |  |
| **Europe** | *Bubalus bubalis* | Czech Republic | n PCR/ seq. | N.S. | 8 | 1 | 13 | ospC and flagellin | *Borrelia burgdorferi* sensu lato | Hrnková et al. [118] |
|  | *Bubalus bubalis* | Hungary | PCR | 2013- 2014 | 60 | 0 | 0 | 18S rRNA | *Babesia/Theileria spp.* | Hornok et al. [119]* |
|  | *Bubalus bubalis* | Hungary | qPCR | 2013- 2014 | 60 | 0  0  0 | 0  0  0 | msp2  23S rRNA  gltA | *Anaplasma Phagocytophilum*  *Rickettsia helvetica*  Other *Rickettsiae* | Hornok et al. [120] |
|  | *Bubalus bubalis* | Italy | n PCR (aborted fetuses) | 2002- 2005 | 164 | 14 | 17.5 | IS1111 | *Coxiella burnetii* | Perugini et al. [121] |
|  |  |  |  |  |  |  |  |  |  |  |
| **North America** | *Bubalus bubalis* | Cuba | qPCR  sn PCR/ seq. | N.S. | 88 | 46  15 | 52 | mspIβ  msp1α | *Anaplasma marginale* | Obregón et al. [122] |
|  | *Bubalus bubalis* | México | n PCR/seq.  IFAT | 2011- 2012 | 154 | 25  37  63  110  131  93 | 16.2  24  40.9  71.4  85.1  60.4 | cytb | *Babesia bovis*  *B. bigemina*  Mixed  *B. bovis*  *B. bigemina*  Mixed | Romero-Salas et al. [123]* |
|  |  |  |  |  |  |  |  |  |  |  |
| **South America** | *Bubalus bubalis* | Argentina | cELISA  n PCR/ seq. | N.S. | 103 | 21  35 | 20  34 | N.S. | *Babesia bovis* | Ferreri et al. [124] |
|  | *Bubalus bubalis* | Brazil | iELISA | N.S. | 491 | 412 |  | - | *Borrelia burgdorferi* | Corrêa et al. [125] |
|  | *Bubalus bubalis* | Brazil | ELISA  n PCR | 2011 | 542  542  542  271  271  271 | 223  103  97  41  44  24 | 41.2  19  18  15.1  16.2  8.9 | 18s rRNA | *Babesia bovis*  *B. bigemina*  Both species  *B. bovis*  *B. bigemina*  Both species | da Silva et al. [126] |
|  | *Bubalus bubalis* | Brazil | n PCR  ELISA | 2011 | 500 | 27  245 | 5.4  49 | msp-5 | *Anaplasma* *marginale* | da Silva et al. [127] |
|  | *Bubalus bubalis* | Brazil | iELISA | 2012 | 330 | 148 | 44.8 | - | *Borrelia burgdorferi* | da Silva et al. [128] |
|  | *Bubalus bubalis* | Brazil | iELISA | 2011 | 4796 | 3597 | 75 | - | *Borrelia burgdorferi* | da Silva et al. [129] |
|  | *Bubalus bubalis* | Brazil | sn PCR/ seq. | 2011 | 200 | 20 | 10 | msp1 α | *Anaplasma* *marginale* | Silva et al. [130] |
|  | *Bubalus bubalis* | Brazil | PCR/seq. | 2012 | 200 | 15 | 7.5 | msp1 α | *Anaplasma marginale* | Silva et al. [131] |
|  | *Bubalus bubalis* | Brazil | nPCR/ seq. | N.S. | 308 | 13  11  3  0 | 4.2  3.6  0.9  0 | 18S rRNA  ITS | *Theileria* spp.  *T. buffeli, T. orientalis, T. sinensis*  *Babesia bovis*  *B. bigemina*  Mixed | Silveira et al. [132] |
|  | *Bubalus bubalis* | Brazil | PCR/seq. | N.S. | 287 | 1  0 | 0.35  0 | 18S rDNA  msp4 | *Theileria buffeli*/*orientalis*  *Anaplasma marginale* | Abate et al. [133] |
|  | *Bubalus bubalis* | Colombia | sn PCR/ seq. | 2014-2016 | 152 | 20 | 13.1 | msp5  mspIα  msp4 | *Anaplasma marginale* | Jaimes-Dueñez et al. [134] |
|  | *Bubalus bubalis* | Colombia | n PCR/ seq. | 2014-2016 | 152 | 36  10  27 | 23.6  6.5  17.7 | 18S rRNA  Hyp  rap-1 | *Babesia* spp.  *B. bigemina*  *B. bovis* | Jaimes-Dueñez et al. [135] |

# Samples used for studies were either blood for direct detection methods (e.g. PCR, microscopy) or sera for serological assays. Other types of samples were mentioned in parentheses.

* Studies not used for meta-analysis due to reasons

^1^ prevalence in endemic areas; ^2^ prevalence in non-endemic areas

**Abbreviations**: **ama-1**; Apical membrane antigen 1 genes, **rpoB**; β-subunit of RNA polymerase, **CCHFV**; Crimean-Congo haemorrhagic fever virus, **cELISA**; Competitive- enzyme-linked immunosorbent assay, **iELISA**; indirect enzyme-linked immunosorbent assay, s**ELISA**; slide enzyme-linked immunosorbent assay, **cytb**; Cytochrome b, **gltA**; Citrate synthase, **groEL**; ‘heatshock operon’, **IFA**; indirect immunofluorescence assay, **IS1111**; *Coxiella burnetii* transposon-like sequence, **LAT**; latex agglutination test, **MPSP**; Major piroplasm surface protein, **msp1β**; Major surface protein–1β encoding gene, **msp4**; Merozoite surface protein 4,.**rap-1**; Rhoptry-associated protein 1 gene, **RLB**; Reverse Line Blotting, **SSU rRNA**; small subunit ribosomal RNA, **TBPs**; Tick-borne pathogens, **msp-5**; major surface protein 5 gene, **msp-2**; merozoite surface protein 2 gene, **msp1α;**  major surface protein α 1 gene, **18S rRNA;** 18 subunit ribosomal RNA gene, **23S rRNA;** 23 subunit ribosomal RNA gene, **5.8S rRNA;** 5.8 subunit ribosomal RNA gene, **ITS;** internal transcribed spacer gene, **cox;** Cytochrome c oxidase subunit, **com1;** encoding 27-kDa outer membrane protein of *C. burnetii*, **Tams1;** encoding major merozoite piroplasm surface antigen of *Theileria annulata*, **Hyp;** hypothetical protein gene, **MPSA;** Merozoite piroplasm surface antigen gene, **SBP-4;** spherical body protein-4 gene targeting *Babesia bovis*, **SBP-2;** spherical body protein-2 gene targeting *B. bovis*, **n PCR;** nested polymerase chain reaction, **sn PCR;** semi nested polymerase chain reaction.
