# Supplementary figures and images for: Global prevalence and species diversity of tick-borne pathogens in buffaloes worldwide: a systematic review and meta-analysis
Source: Parasit Vectors. 2023 Mar 30;16:115. doi: 10.1186/s13071-023-05727-y (PMC10061416; doi:10.1186/s13071-023-05727-y)

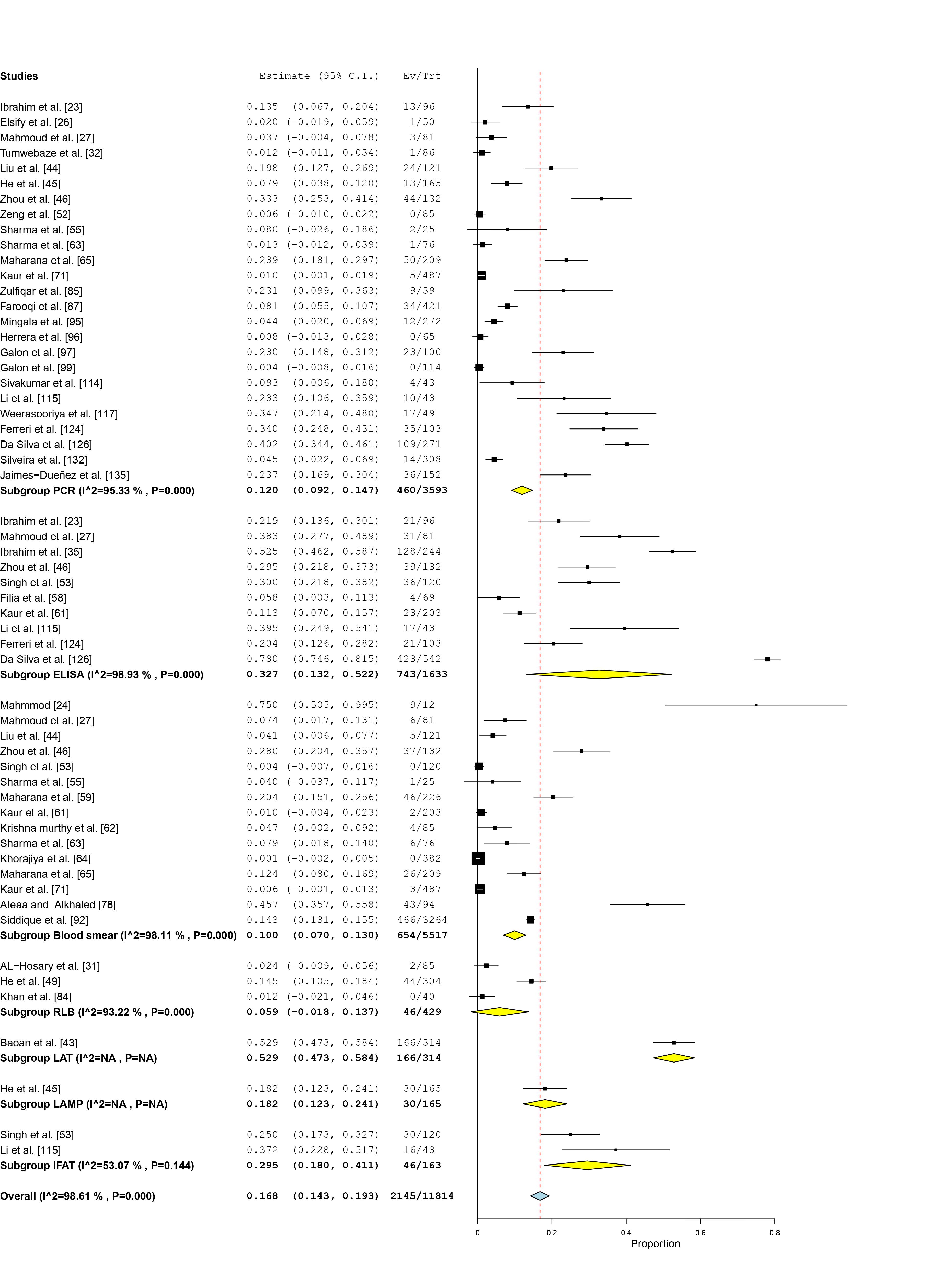

Supplement: Supplementary file 2 — Additional file 2: Figure S1. Forest plot diagrams for random effects in the meta-analysis of the prevalence of Babesia spp. infections in buffaloes worldwide. The middle point of each line indicates the prevalence, while the length of the line is the 95% confidence interval for each study. Diamonds refers to the prevalence in accordance with detection methods. [file 13071_2023_5727_MOESM2_ESM.jpg]

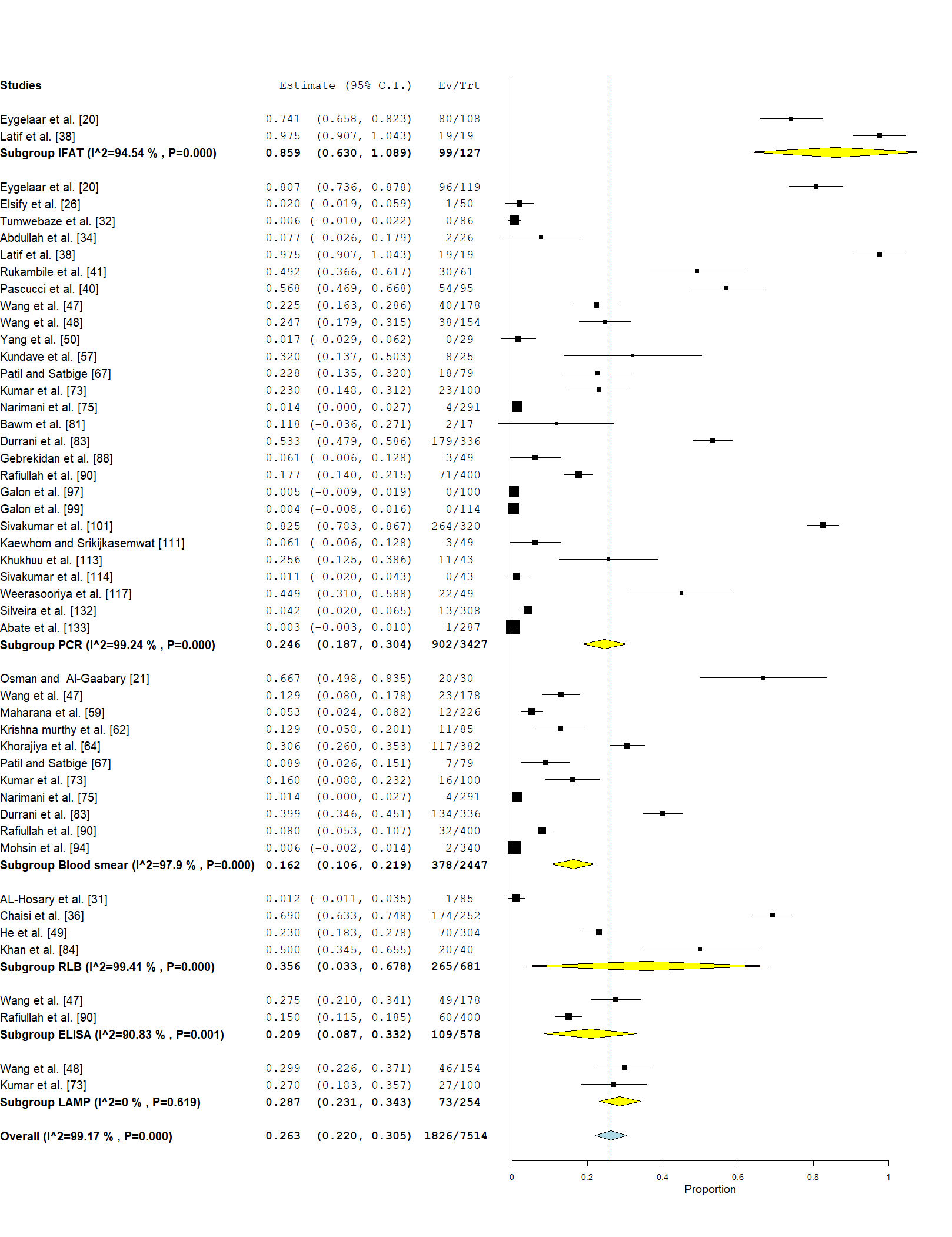

Supplement: Supplementary file 3 — Additional file 3: Figure S2. Forest plot diagrams for random effects in the meta-analysis of the prevalence of Theileria spp. infections in buffaloes worldwide. The middle point of each line indicates the prevalence, while the length of the line is the 95% confidence interval of each study. Diamonds refers to the prevalence in accordance with detection methods. [file 13071_2023_5727_MOESM3_ESM.png]

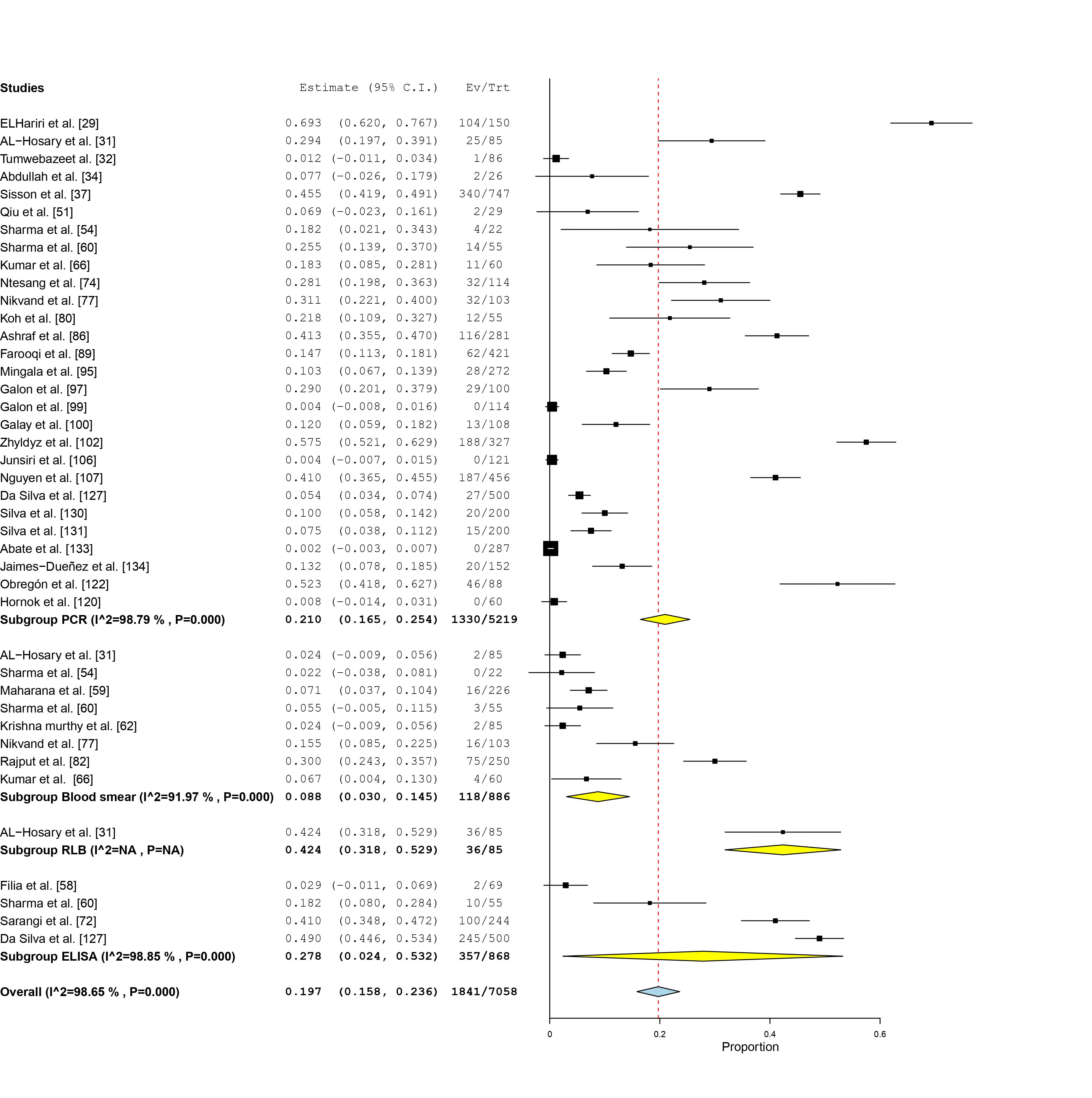

Supplement: Supplementary file 4 — Additional file 4: Figure S3. Forest plot diagrams for random effects in the meta-analysis of the prevalence of Anaplasma spp. infections in buffaloes worldwide. The middle point of each line indicates the prevalence, while the length of the line is the 95% confidence interval of each study. Diamonds refers to the prevalence in accordance with detection methods. [file 13071_2023_5727_MOESM4_ESM.jpg]
